# Pentastomids of wild snakes in the Australian tropics^☆^

**DOI:** 10.1016/j.ijppaw.2013.12.003

**Published:** 2013-12-31

**Authors:** Crystal Kelehear, David M. Spratt, Denis O’Meally, Richard Shine

**Affiliations:** a School of Biological Sciences, A08, University of Sydney, NSW 2006, Australia; b Australian National Wildlife Collection, CSIRO Ecosystem Sciences, GPO Box 1700, Canberra, ACT 2601, Australia; c Institute of Applied Ecology, University of Canberra, ACT 2601, Australia

**Keywords:** Colubrid, Elapid, Python, *Raillietiella orientalis*, *Waddycephalus*, Zoonosis

## Abstract

Pentastomids are endoparasites of the respiratory system of vertebrates, maturing primarily in carnivorous reptiles. Adult and larval pentastomids can cause severe pathology resulting in the death of their intermediate and definitive hosts. The study of pentastomids is a neglected field, impaired by risk of zoonoses, difficulties in species identification, and life cycle complexities. We surveyed wild snakes in the tropics of Australia to clarify which host species possess these parasites, and then sought to identify these pentastomids using a combination of morphological and molecular techniques. We detected pentastomid infections in 59% of the 81 snakes surveyed. The ubiquity of pentastomid infections in snakes of the Australian tropics sampled in this study is alarmingly high considering the often-adverse consequences of infection and the recognized zoonotic potential of these parasites. The pentastomids were of the genera *Raillietiella* and *Waddycephalus* and infected a range of host taxa, encompassing seven snake species from three snake families. All seven snake species represent new host records for pentastomids of the genera *Raillietiella* and/or *Waddycephalus*. The arboreal colubrid *Dendrelaphis punctulatus* and the terrestrial elapid *Demansia vestigiata* had particularly high infection prevalences (79% and 100% infected, respectively). *Raillietiella orientalis* infected 38% of the snakes surveyed, especially frog-eating species, implying a frog intermediate host for this parasite. *Raillietiella orientalis* was previously known only from Asian snakes and has invaded Australia via an unknown pathway. Our molecular data indicated that five species of *Waddycephalus* infect 28% of snakes in the surveyed area. Our morphological data indicate that features of pentastomid anatomy previously utilised to identify species of the genus *Waddycephalus* are unreliable for distinguishing species, highlighting the need for additional taxonomic work on this genus.

## Highlights

• Pentastomids infected 59% of snakes surveyed in the Australian tropics.
• Pentastomids were of the genera *Raillietiella* and *Waddycephalus*.
• *Raillietiella orientalis* has invaded Australia via an unknown pathway.
• Five species of *Waddycephalus* were present in the Australian tropics.
• Morphological features are unreliable for distinguishing *Waddycephalus* species.

## Graphical abstract

**Figure:**
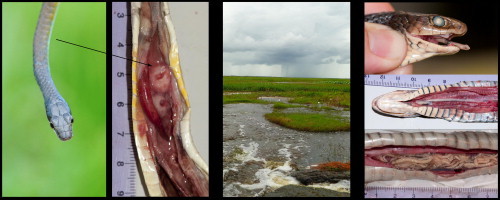

## 1 Introduction

Pentastomids are long-lived endoparasites of the respiratory system of vertebrates, and are arguably the oldest metazoan parasites known to science. Prehistoric larvae closely resembling extant primary larvae appeared in the fossil record approximately 100 million years prior to the vertebrates they now parasitize (Self, 2009). Pentastomids mature primarily in carnivorous reptiles (90% of pentastomid species mature in snakes, lizards, crocodiles, and turtles), but also infect toads, birds (seabirds and vultures), and mammals (canines, felines, reindeer, sugar gliders, and humans; Paré, 2008). Pentastomids generally have an indirect life cycle, utilising at least one intermediate host; suitable intermediate hosts for pentastomids span diverse taxa (*e.g.*, mammals, reptiles, insects, fish, and amphibians) but for most species the intermediate host is unknown (Riley, 1986; Paré, 2008). Larval pentastomids enter their definitive host when it consumes an infected intermediate host. These larvae tunnel out of the digestive system and through their definitive host to the lungs, generating lesions and scars along their migration path (Jacobson, 2007). In intermediate or accidental hosts these larvae can establish widespread visceral infections (*e.g.*, Brookins et al., 2009; Haddadzadeh et al., 2010; Mätz-Rensing et al., 2012; Yakhchali and Tehrani, 2012). In humans, pentastomiasis is most commonly caused by *Linguatula serrata* or *Armillifer armillatus* (Paré, 2008); these parasites may be transmitted via food or water contaminated with their eggs, or in the case of *A. armillatus*, particularly via consumption of undercooked snake flesh (*e.g.*, Yapo Ette et al., 2003; Lai et al., 2010; Ibinaiye et al., 2011; Hardi et al., 2013). Adult pentastomids feed primarily on blood from host capillary beds in the lungs and can cause severe pathology resulting in death (Paré, 2008). Adult pentastomids reach large body sizes (up to 15 cm), physically occluding respiratory passages and inducing suffocation. The two pairs of hooks they use for attaching to lung tissue can cause perforations and haemorrhaging, and degrading moulted cuticles shed into the lung lumen by growing pentastomids may induce putrid pneumonia (Jacobson, 2007).

The class Pentastomida comprises two orders: Cephalobaenida and Porocephalida, both of which are represented in Australia (Self, 2009). Within the order Cephalobaenida is the family Cephalobaenidae, that contains the largest genus of pentastomids: *Raillietiella,* comprised of ∼39 species and known from all continents where reptiles occur. Raillietiellids are small pentastomids (generally <25 mm) that mature primarily in the lungs of reptiles; most commonly, snakes and small lizards serve as definitive hosts (Ali et al., 1985). For the only raillietiellid where the life cycle has been experimentally elucidated, the eggs shed by infected definitive hosts are consumed by coprophagous insect intermediate hosts, develop to the infective stage, and are then eaten by the definitive host, thus completing the life cycle (Ali and Riley, 1983). For raillietiellid definitive hosts that do not eat insects, intermediate hosts may be snakes, lizards, and/or amphibians (Ali et al., 1982). Several species of *Raillietiella* are known in Australia: *Raillietiella amphiboluri* in the dragon *Pogona barbata* (Ali et al., 1985); *Raillietiella frenata* (*=R. frenatus*) in the toad *Rhinella marina*, the treefrog *Litoria caerulea*, and the geckos *Hemidactylus frenatus* (Kelehear et al., 2011) and *Gehyra australis* (Barton, 2007), and *Raillietiella scincoides* in the skink *Tiliqua scincoides* (Ali et al., 1984) and the gecko *Nephrurus laevissimus* (Bursey and Goldberg, 1999). An additional species, *Raillietiella orientalis*, previously known only from Asian snakes (Ali et al., 1982), was recently recorded in the lungs of two individual *R. marina* in the Northern Territory, but toads were considered an incidental host in this instance (Kelehear et al., 2011). Mature unidentified raillietiellids have also been reported in two Australian snake species, the elapids *Pseudechis australis* and *Pseudonaja textilis* (Riley and Spratt, 1987). Within the order Porocephalida is the family Sambonidae that contains the genus *Waddycephalus*, comprised of 10 species known from Asian, Fijian, and Australian snakes (Riley and Self, 1981b). Seven species of *Waddycephalus* have been recorded in Australia, occurring across a vast geographic range from Tasmania to Cape York, and infecting several taxa (elapids, pythons and colubrids) of snakes (Riley and Self, 1981b). Six of these seven species were first described in 1981, highlighting the need for taxonomic work on this genus – yet more than thirty years have passed with no further research published on this topic, except new host and intermediate host records (see Riley et al., 1985; Riley and Spratt, 1987).

The study of pentastomids has been neglected because of the risk of zoonoses, difficulties in species identification (see Kelehear et al., 2011), and life cycle complexity (involving at least one intermediate host) hampering experimental manipulation. Most pentastomid research concerns evolutionary classification (reviewed in: Waloszek et al., 2006), taxonomy, new species descriptions, new host reports, records of prevalence and intensity, and occasional veterinary and clinical case studies on pathology or death due to pentastomiasis (Paré, 2008). Considering the often adverse consequences of infection in captive and wild hosts, the zoonotic potential of these parasites, and the possibility that these parasites are being inadvertently introduced to Australia (Barton, 2007; Kelehear et al., 2011, 2013), we urgently need data on the identity of these parasites, the prevalence and intensity of infections, and the host species that are likely to be infected. However, delineating species of parasites using morphological criteria alone can be difficult (Criscione et al., 2005), and can often lead to misidentifications, particularly in taxa with few or variable distinguishing morphological characters (*e.g.*, Kelehear et al., 2011). Here we combine traditional analyses of morphological appearance with molecular analyses to clarify the species of pentastomids infecting wild snakes in the Australian tropics.

## 2 Materials and methods

### 2.1 Snake collection and dissection

Road-killed snakes were collected on roads surrounding Middle Point (12°37′S, 131°18′E) in the tropics of the Northern Territory, Australia, between November 2008 and July 2011. Only snakes that were freshly killed with relatively intact airways were collected. Snakes were stretched straight along a ruler to measure total length from snout to tail tip (STL) to the nearest cm. *Demansia* species were distinguished by ventral scale counts following the procedure outlined in Dowling (1951); specimens with ⩽197 ventral scales were identified as *D. vestigiata* and those with ⩾198 ventral scales were identified as *Demansia papuensis* (as per, Shea, 1998). The mouth, trachea, bronchi, lungs, and air sac were inspected for pentastomids. All pentastomids were removed and placed immediately into cold 70% ethanol.

### 2.2 Pentastome DNA extraction, amplification, and analyses

We sequenced the mitochondrial *cytochrome C oxidase subunit 1* (*COX1*) for 40 pentastomes from snakes and included in analyses three additional sequences (two from the snake *Stegonotus cucullatus* and one from the cane toad *R. marina*) that have been published previously (Kelehear et al., 2011). Ethanol-preserved samples were first air-dried and then extracted using the Chelex method as described previously in Kelehear et al. (2011). For some samples that failed to yield products from the Chelex extraction method, we extracted total DNA using a modified salting out method (Cawthorn et al., 2011). Briefly, a small sample of an ethanol-preserved specimen was first air-dried and then incubated overnight at 56 °C in 100 μL of TEN buffer (40 mM Tris/HCl; 100 mM NaCl; 20 mM EDTA, pH 7.2), 1% SDS and 0.2 mg/mL of proteinase K. To this, 75 μL of ammonium acetate was added and the sample chilled at −80 °C for 10 min before spinning at maximum speed in a microcentrifuge. The supernatant was drawn off and added to 2 volumes of ethanol, chilled and centrifuged as before and the supernatant discarded. The pellet was washed twice in 70% ethanol and resuspended in 50 μL of ddH_2_0. Purified DNA (1–2 μL) was used in PCR reactions, and the remainder stored at −20 °C. PCR amplifications were performed as described previously in Kelehear et al. (2011). Forward and reverse sequence reactions were performed by Macrogen Inc. (Seoul, Korea) and aligned using Mega 5.10 (Tamura et al., 2011) and by eye. Regions of ambiguous alignment were excluded and gaps were treated as missing. We constructed a neighbour joining dendrogram, using Kimura 2-parameter (K2P) distances and tested the tree topology with 1000 bootstrap replicates.

### 2.3 Pentastome morphology

To visualise diagnostic characteristics, pentastomids were cleared in lactophenol, then sufficiently small specimens were mounted on slides and cover-slipped to hold them flat. Larger specimens were dissected to better visualise their anatomical features: their heads were removed, the cephalic end was flattened and the hooks were dissected out, generally from one side of the body only. All specimens measured were either males with fully formed copulatory spicules or females containing eggs. Body length was measured from the tip of the head to the end of the caudal segment; body width was measured at the widest point. When specimens were intact, counts of annuli (total number and number of post-vaginal annuli; see Riley and Self, 1981b) were made under the 10X objective lens of a compound microscope. Dimensions of hooks from raillietiellids were measured as distances from the hook tip to the inside corner of the anterior fulcrum (AB: barb length), and from the back corner of the anterior fulcrum to the outside corner of the posterior fulcrum (BC: overall length; see Fig. 7 in Kelehear et al., 2011). For comparison with other raillietiellids, we extracted raw measurements of AB/BC of posterior hooks from Fig. 3 in Ali et al. (1982). Unfortunately, Ali et al. (1982) did not provide corresponding body size data. Lengths of raillietiellid copulatory spicules were measured along the centre-line of the spicule from start to base; width of copulatory spicules was measured at the widest point of the base. Where possible, all four hooks and both copulatory spicules were measured, though for larger pentastomids only one spicule could be measured. Hook dimensions for *Waddycephalus* spp. were measured as per Riley and Self (1981b). We extracted raw hook measurements of AD/BC for *Waddycephalus* spp. from Figs. 6 and 11 in Riley and Self (1981b) to enable direct comparison of our specimens; again, corresponding body size data were unavailable for most specimens.

### 2.4 Terminology

Our terminology follows that of Bush et al. (1997). We report prevalence as the number of hosts infected with pentastomids divided by the total number of hosts inspected. We report intensity as the number of individual pentastomids within infected hosts.

### 2.5 Data analysis

To examine the influence of host species on pentastome prevalence and intensity, we included only those host taxa where sample sizes were >7 (*Acanthophis praelongus*, *D. vestigiata*, *D. punctulatus*, *Liasis fuscus*, *S. cucullatus*, *Tropidonophis mairii*). We applied square root transformations to our count data (pentastome intensity) prior to analyses but we report raw data in the text, tables, and figures. Analyses of pentastome morphology were performed on mean values for all measurements taken on paired structures (anterior hooks, posterior hooks, and copulatory spicules). Analyses were based on measurements of only one paired structure in large and poorly cleared specimens where it was not possible to measure all structures. For each aspect of pentastome body size (length and width) we performed one-way ANOVAs with pentastome sex and host species as independent variables. For each aspect of pentastome hook morphology (AB and BC) we performed one-way ANOVAs with pentastome body size, sex, and host species as independent variables. We included only those host species for which we had obtained measurements from >5 pentastomids. We used Tukey’s HSD post hoc tests to determine where the differences lay. All analyses were performed in JMP Pro 9.0 (SAS, 2010) with alpha set at <0.05.

## 3 Results

### 3.1 Prevalence and intensity of infections

**Fig. 1:**
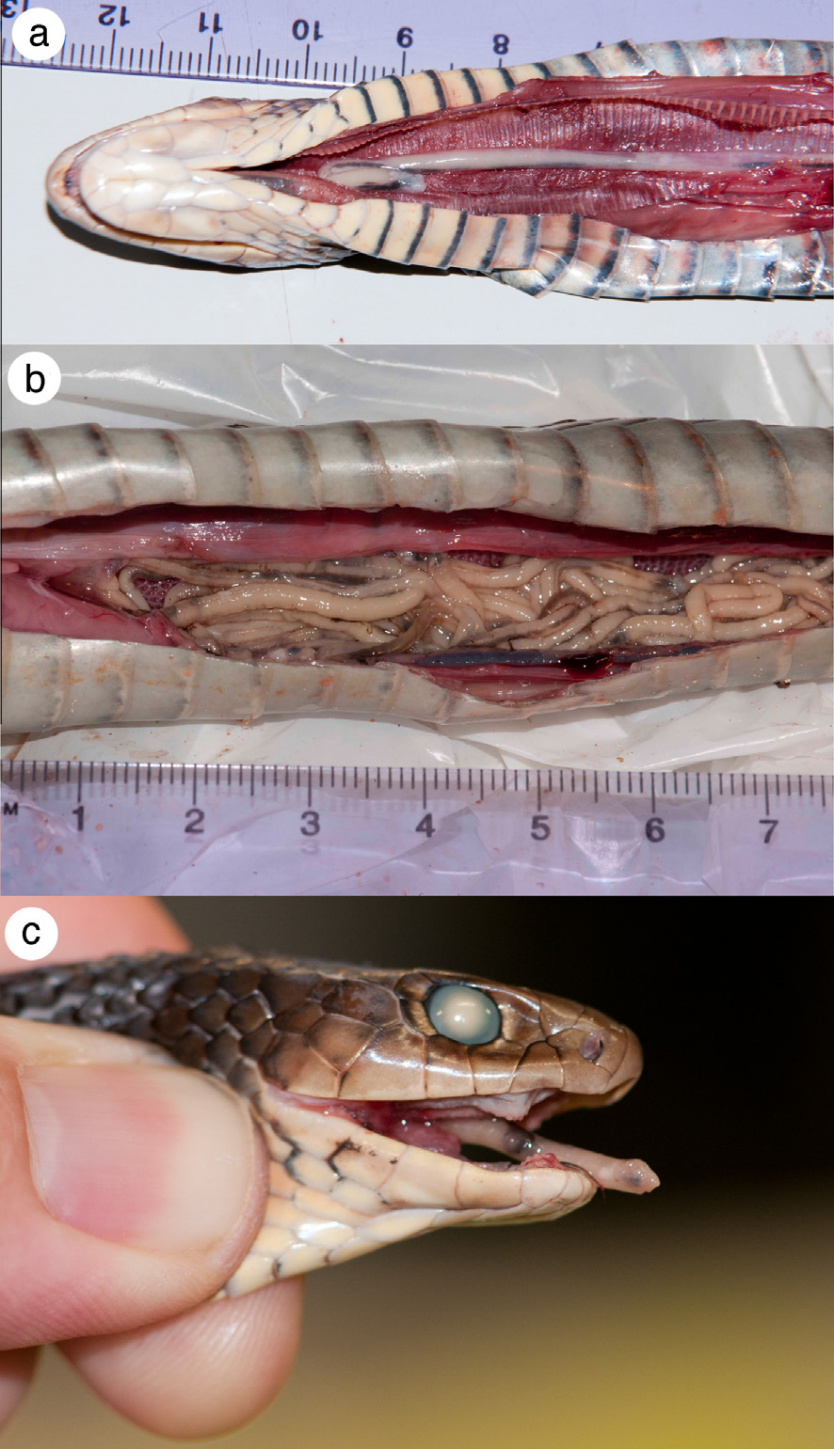
*Raillietiella orientalis* in road-killed lesser black whip snakes, *Demansia vestigiata*, collected in the Northern Territory of Australia. (a) Dissected trachea containing a single long *R. orientalis*; (b) Severe occlusion of the lung by 68 *R. orientalis*; (c) *R. orientalis* crawling out of the mouth of a snake. All scale rules are in centimetres; all photo credits to Crystal Kelehear.

**Fig. 2:**
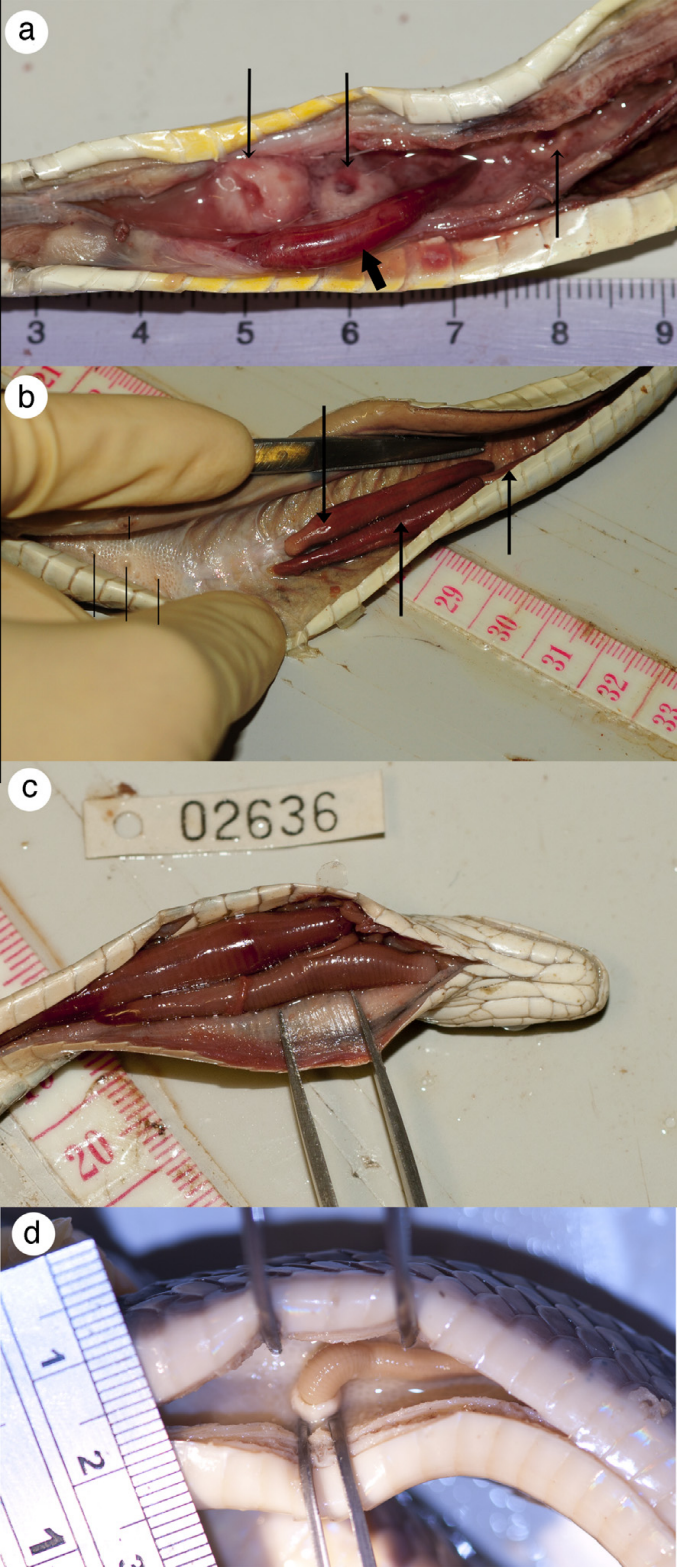
*Waddycephalus* spp. in the lungs of road-killed snakes collected in the Northern Territory of Australia. (a) One female *Waddycephalus* sp. (short arrow) and three lesions surrounding *Waddycephalus* attachment sites (long arrows) in the dissected lung of a green tree snake, *Dendrelaphis punctulatus*; (b) Three (one is partially obscured) female *Waddycephalus* sp. (arrows), in the dissected lung of *D. punctulatus*. Note also multiple visible attachment sites (lines); (c) Two large female and several small male *Waddycephalus* sp. filling the trachea of the same *D. punctulatus* depicted in (b); (d) One *Waddycephalus* sp. deeply embedded in the lung tissue of an ethanol-preserved slaty-grey snake, *Stegonotus cucullatus*. All scale rules are in centimetres; all photo credits to Crystal Kelehear.

We dissected 81 snakes of 10 different species, and recovered pentastomids from 48 of these snakes (Table 1). These infections comprised a total of 359 individual pentastomids from the genera *Raillietiella* and *Waddycephalus* (see species identifications below). Most snakes contained pentastomids of only one genus (25 snakes were infected with *Raillietiella* only, 17 were infected with *Waddycephalus* only) but six snakes had infections of both genera. Pentastomids were recovered from the mouth, trachea, lungs, and air sacs of dissected snakes (Figs. 1 and 2). Large lesions (Fig. 2a, and b) surrounded the mouthparts of *Waddycephalus* spp. where they were embedded in lung tissue (Fig. 2d); in some cases these lesions were present even in the absence of current *Waddycephalus* infections, likely indicating prior infection with this pentastomid.

*Raillietiella orientalis* were recovered from six snake species: the colubrids *D. punctulatus* and *T. mairii*, the elapids *A. praelongus*, *D. papuensis*, and *D. vestigiata*, and the python *L. fuscus* (Table 1). Overall, 31 of 81 snakes were infected with *R. orientalis*; infection intensity ranged from 1 to 77 (mean ± S.E. = 9.1 ± 3.2). The likelihood of being infected with *R. orientalis* varied among host species (*χ*^2^_5_ = 55.10, *P* < 0.0001) but did not vary with host body length (*χ*^2^_1_ = 0.10, *P* = 0.75). *Demansia vestigiata* were more likely to be infected than were any other snake species. *Demansia vestigiata* was the only host species for which we had sufficient data to analyse whether the intensity of *R. orientalis* infections vary with host body size. Larger snakes tended to have more *R. orientalis*, but this relationship fell short of statistical significance (*F*_1,17_ = 3.76, *P* = 0.069).

*Waddycephalus* spp. were recovered from five snake species: the colubrids *D. punctulatus*, *S. cucullatus*, and *T. mairii*, and the elapids *A. praelongus* (one nymphal *Waddycephalus* only) and *D. vestigiata* (Table 1). Overall, 23 of 81 snakes were infected with *Waddycephalus* spp.; infection intensity ranged from 1 to 10 (mean ± S.E. = 3.4 ± 0.6; Table 1). The likelihood of being infected with *Waddycephalus* sp. varied with host species (*χ*^2^_5_ = 27.21, *P* < 0.0001) but not with host body length (χ^2^_1_ = 0.01, *P* = 0.91). *D. punctulatus* and *S. cucullatus* were significantly more likely to be infected with *Waddycephalus* sp. than were any other snake species, but the two host species did not differ from one another in infection likelihood.

### 3.2 Molecular data

**Fig. 3:**
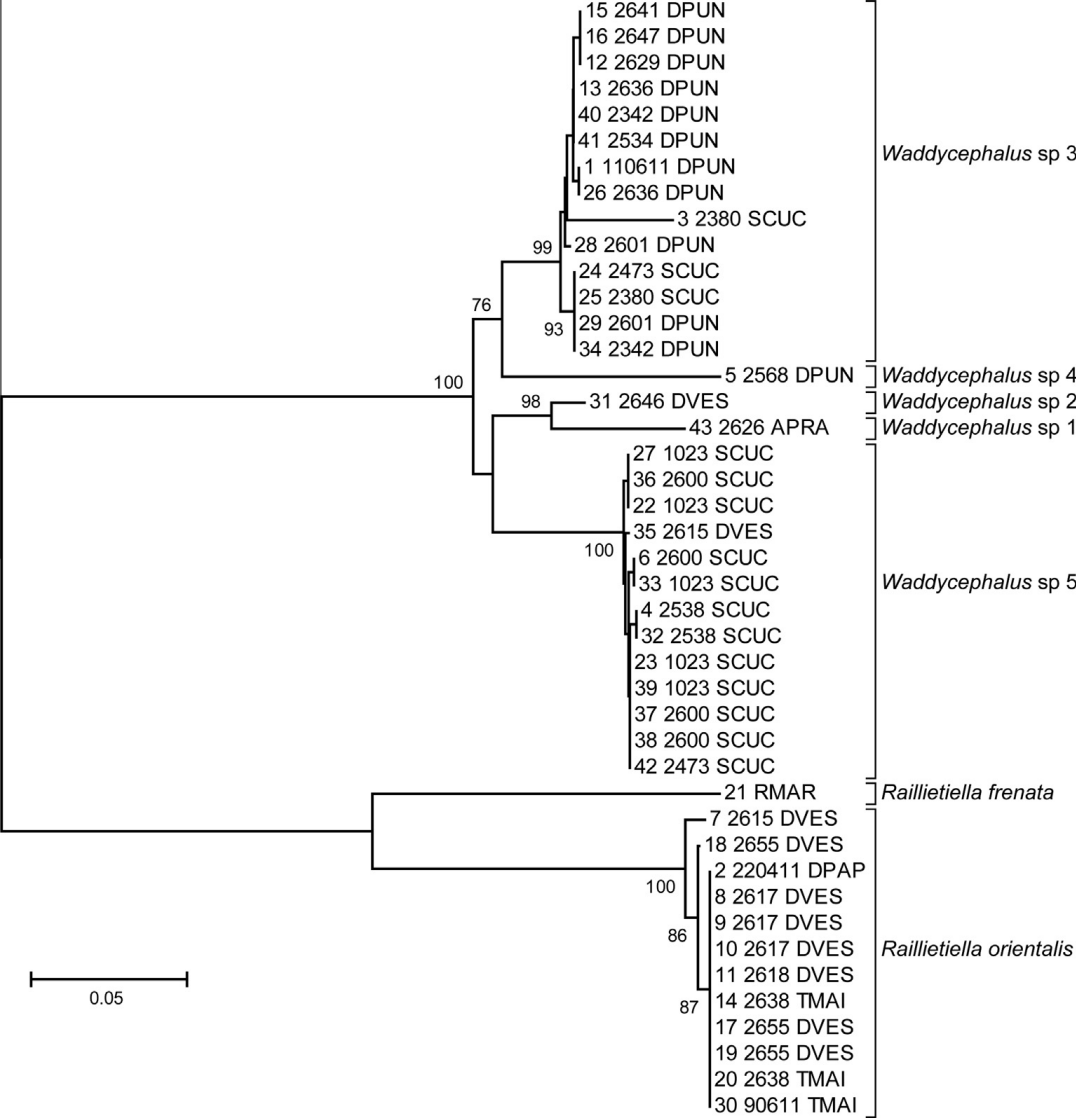
Neighbour joining topology of *Waddycephalus* and *Raillietiella* species, calculated from K2P distances. We included partial *COX1* sequences from 43 pentastomids. Host species identifications follow abbreviations given in the text. Branch lengths are proportional, and the scale bar represents 0.05 base substitutions per site. Bootstrap support values greater than 70 are shown next to the branches (1000 replicates). All pentastomids were from Australian snakes except *R. frenata* which was from the cane toad, *Rhinella marina*.

We analyzed sequences from 43 pentastomids: 13 raillietiellids, taken from seven snakes (4 × *D. vestigiata* [*DVES*], 1 × *D. papuensis* [*DPAP*], 2 × *T. mairii* [*TMAR*]), and one toad (*R. marina*: *RMAR*); and 30 individual *Waddycephalus* spp. taken from 17 individual snakes (9 × *D. punctulatus* [*DPUN*], 5 × *S. cucullatus* [*SCUC*], 2 × *D. vestigiata*, 1 × *A. praelongus* [*APRA*]; Fig. 3). Forty sequences were new for this study (GenBank accession numbers: KF885745–KF885783, KF908012), and three were already available (GenBank accession numbers: JF975594–6). After excluding regions of ambiguous alignment and gaps, the complete data set consisted of 486 bp of *COX1* sequence.

Our data suggest that one species of raillietiellid (*R. orientalis*) and five species of *Waddycephalus* are present in the snakes examined (Fig. 3). Of the 30 *Waddycephalus* that we sequenced, three species were recorded from single snake specimens (sp. 1: *A. praelongus*, sp. 2: *D. vestigiata*, and sp. 4: *D. punctulatus*); sp. 3 primarily infected *D. punctulatus* and, to a lesser extent, *S. cucullatus*; sp. 5 primarily infected *S. cucullatus* but was also recovered from one *D. vestigiata* (Fig. 3). Our molecular data suggest that each *Waddycephalus* infection (within an individual snake) was generally comprised of a single *Waddycephalus* species: for the eight snakes from which we sequenced multiple pentastomids, only one snake (2473 SCUC) possessed two species of *Waddycephalus* (sp. 3 and 5).

To assess within and between species divergence, we calculated a matrix of mean K2P genetic distances (Table 2) and a neighbor joining dendrogram (Fig. 3). Within species for which there was more than one sample, K2P distances were low (0.003 for *Waddycephalus* sp. 5 and *R. orientalis*; 0.010 for *Waddycephalus* sp. 3). Between *Waddycephalus* species, the K2P distances ranged from 0.053 substitutions per site between sp. 1 and sp. 2, to 0.159 between sp. 1 and sp. 4; between the two species of *Raillietiella*, one from snakes (this study) and one from toads (Kelehear et al., 2011), the genetic distance was 0.212 (Table 2). The mean K2P distance between *Waddycephalus* and *Raillietiella* was 0.422 substitutions per site.

### 3.3 Morphological data

#### 3.3.1 *Raillietiella orientalis*

We identified raillietiellids as *R. orientalis* based on the distinctive morphology of their large, flared, and highly ornamented copulatory spicules (see Ali et al., 1982; Kelehear et al., 2011). We measured 31 adult (15 female, 16 male) *R. orientalis* from a total of four snake species (Table 3). Specimens of *R. orientalis* were cylindrical to fusiform in shape and were generally long and thin (Fig. 1). Body length ranged from 4.0 to 65.2 mm, and width from 0.19 to 2.37 mm (Table 3). Pentastome length differed between males and females (*F*_1,28_ = 60.31, *P* < 0.0001): females were longer than males (LS means = 35.61 *vs* 5.45). Raillietiellids infecting *D. vestigiata* were significantly longer than those infecting *T. mairii* and *A. praelongus* (*F*_2,25_ = 15.41, *P* < 0.0001). Pentastome body width did not vary with pentastome sex (*F*_1,28_ = 1.72, *P* = 0.20).

**Fig. 4:**
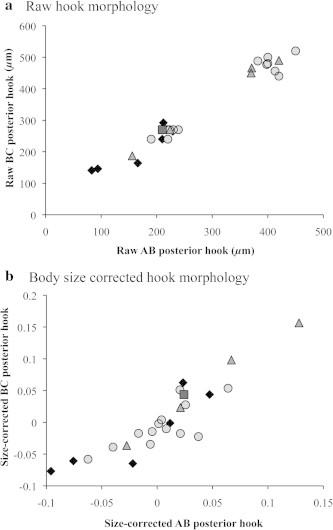
Posterior hook morphology of *Raillietiella orientalis*, collected from snakes in the Northern Territory of Australia. Barb length (AB) *versus* overall length (BC) of pentastomids measured from four snake species: Northern death adder (*Acanthophis praelongus*; black diamonds), lesser black whip snake (*Demansia vestigiata*; grey circles), keelback (*Tropidonophis mairii*; grey triangles), and water python (*Liasis fuscus*; dark grey squares). (a) Raw hook measurements uncorrected for pentastome body size, note potential species clusters; (b) Hook measurements corrected for pentastome body size (residual scores from a linear regression of hook measurements against pentastome body size), note that clusters now disappear.

**Fig. 5:**
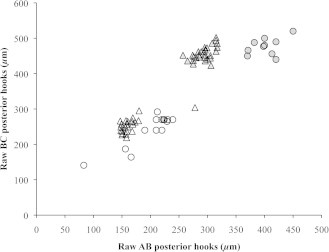
Comparison of posterior hook morphology of *Raillietiella orientalis* from the Northern Territory of Australia *versus* Asia. Raw barb length (AB) *versus* overall length (BC) of posterior hooks of *R. orientalis* measured in the current study (circles) and in Ali et al., 1982 (triangles). Gray symbols denote female pentastomes and open symbols denote male pentastomes.

All anterior hooks of *R. orientalis* were sharp, as is characteristic of pentastomids in the genus *Raillietiella*. Hook size data are given in Table 3. We first performed one-way ANOVAs with pentastomid body length as a continuous independent variable and hook morphology (AB, BC of anterior and posterior hooks) as the dependent variable. Hook size was positively correlated with body length in all cases (*P* < 0.0001 for each analysis). We then conducted more thorough analyses correcting for pentastome length, sex, and host species. AB of anterior hooks did not vary significantly among host species (*F*_2,17_ = 0.06, *P* = 0.94) or change with pentastome body length (*F*_1,17_ = 1.55, *P* = 0.23), but male pentastomes had shorter anterior hook AB lengths than did females (*F*_1,17_ = 4.53, *P* = 0.048). BC of anterior hooks did not vary significantly with host species (*F*_2,17_ = 0.11, *P* = 0.89), pentastome body length (*F*_1,17_ = 2.11, *P* = 0.16), or pentastome sex (*F*_1,17_ = 3.19, *P* = 0.09). AB of posterior hooks did not vary significantly among host species (*F*_2,17_ = 0.18, *P* = 0.83), or change with pentastome body length (*F*_1,17_ = 2.83, *P* = 0.11), but male pentastomes had shorter posterior hook AB lengths than did females (*F*_1,17_ = 5.04, *P* = 0.038). BC of posterior hooks did not vary with host species (*F*_3,17_ = 0.01, *P* = 0.99, Fig. 4), pentastome length (*F*_1,17_ = 3.63, *P* = 0.07) or pentastome sex (*F*_1,17_ = 4.41, *P* = 0.05, Fig. 5). Male copulatory spicules were strongly ornamented and flared at the base and were 900–1600 μm long, and 371–1500 μm wide (Table 3). The mean length and width of copulatory spicules did not differ with host species (*F*_1,7_ = 4.62, *P* = 0.07, and *F*_1,7_ = 1.03, *P* = 0.34, respectively) or pentastome length (*F*_1,7_ = 3.76, *P* = 0.09, and *F*_1,7_ = 2.19, *P* = 0.17, respectively).

In keeping with methods employed in previous studies of pentastomid morphology, we first plotted AB (barb length) against BC (overall length) of posterior hooks to visualize distinct clusters indicative of separate species (Fig. 4a). Measurements grouped into two distinct clusters, implying two separate species (Fig. 4a). However, when we included body size as a covariate, these discrete clusters disappeared, implying a single species (Fig. 4b). We compared our raw measurements of AB and BC of posterior hooks with those taken on 52 *R. orientalis* measured by Ali et al. (1982). Overall, AB of posterior hooks differed with pentastome sex (*F*_1,73_ = 323.48, *P* < 0.0001) and with study (*F*_1,73_ = 50.66, *P* < 0.0001; Fig. 5). Measurements of AB were significantly larger in female *vs* male pentastomes and in our study *vs* in Ali et al. (1982). BC of posterior hooks also varied with pentastome sex (*F*_1,73_ = 651.62, *P* < 0.0001) but did not differ between the two studies (*F*_1,73_ = 0.59, *P* = 0.4493; Fig. 5). Data on relative hook size has recently been shown to be important in eliminating false species clusters (Kelehear et al., 2011); unfortunately, raw body size data were not presented in Ali et al. (1982), precluding any comparison of relative hook sizes between studies.

#### 3.3.2 *Waddycephalus* spp.

Pentastomids of the genus *Waddycephalus* sp. are large, bright red, and cylindrical (Fig. 2). We measured 38 *Waddycephalus* spp. (18 female, 11 male, 2 nymphs) from a total of five snake species (Table 4). *Waddycephalus* adult body length ranged from 5.3 to 42.0 mm, and width from 0.3 to 5.5 mm (Table 4). Body length and width were positively correlated (*F*_1,21_ = 78.91, *P* < 0.0001); female pentastomes were longer than males (*F*_1,17_ = 27.34, *P* < 0.0001; LS means = 27.4 *vs* 9.9) but there was no significant difference in relative body width (corrected for body length) between sexes (*F*_1,16_ = 1.17, *P* = 0.29).

As with raillietiellids, hook morphology is important for identifying pentastomids of the genus *Waddycephalus* (Riley and Self, 1981b). AD of anterior hooks and posterior did not vary with pentastome body length (*F*_1,14_ = 0.007, *P* = 0.94, and *F*_1,13_ = 0.03, *P* = 0.86, respectively), but male pentastomes had shorter anterior and posterior hook AD lengths than did females (*F*_1,14_ = 6.67, *P* = 0.02, and *F*_1,13_ = 7.63, *P* = 0.02, respectively; Table 4). BC of anterior and posterior hooks did not vary with pentastome body length (*F*_1,14_ = 0.11, *P* = 0.75, and *F*_1,13_ = 0.15, *P* = 0.70, respectively), or pentastome sex (*F*_1,14_ = 3.44, *P* = 0.08, and *F*_1,13_ = 3.02, *P* = 0.11, respectively; Table 4).

**Fig. 6:**
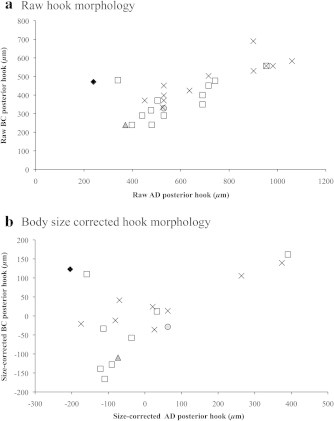
Posterior hook morphology of *Waddycephalus* spp., collected from snakes in the Northern Territory of Australia. Overall hook length (AD) *versus* depth of hook shank (BC) of posterior hooks in *Waddycephalus* of the Northern death adder (*Acanthophis praelongus*; black diamond), lesser black whip snake (*Demansia vestigiata*; grey circle), keelback (*Tropidonophis mairii*; grey triangle), green tree snake (*Dendrelaphis punctulatus*; open squares), and slaty grey snake (*Stegonotus cucullatus*; crosses). (a) Raw hook measurements uncorrected for pentastome body size, note four potential clusters; (b) Hook measurements corrected for pentastome body size (residual scores from a linear regression of hook measurements against pentastome body size), note that clusters realign.

**Fig. 7:**
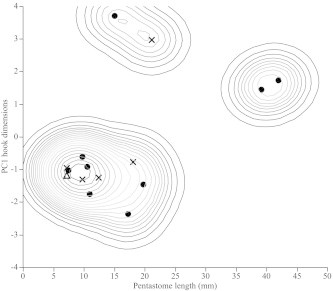
*Waddycephalus* spp. body lengths *versus* the first principal components (PC1) of a principal components analysis of hook dimensions (anterior AD, anterior BC, posterior AD, posterior BC). Contour lines show quantile contours in 5% intervals from a nonparametric density analysis. Different genetic species groupings are denoted by different symbols (triangle = *Waddycephalus* sp. 2; circles = *Waddycephalus* sp. 3; crosses = *Waddycephalus* sp. 5; body size data was not attained for *Waddycephalus* sp. 1 or 4).

**Fig. 8:**
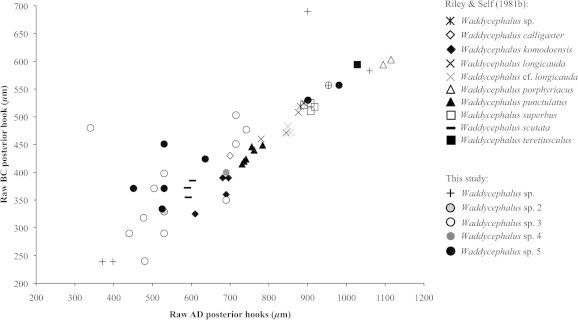
Morphology of pentastomids in the current study (*Waddycephalus* sp. 2–5) compared with previously described species measured in Riley and Self (1981b). Overall hook length (AD) *versus* depth of hook shank (BC) of posterior hooks of *Waddycephalus* sp. (current study: plus signs), *Waddycephalus* sp. 2 (pale grey circle), *Waddycephalus* sp. 3 (open circles), *Waddycephalus* sp. 4 (dark grey circle), *Waddycephalus* sp. 5 (closed circles), *Waddycephalus* sp. (Riley and Self (1981b): asterisks), *Waddycephalus calligaster* (open diamond), *Waddycephalus komodoensis* (closed diamonds), *Waddycephalus longicauda* (black crosses), *Waddycephalus* cf. *longicauda* (grey crosses), *Waddycephalus scutata* (black dashes), *Waddycephalus superbus* (open squares), *Waddycephalus teretiusculus* (closed square), *Waddycephalus porphyriacus* (open triangles), and *Waddycephalus punctulatus* (closed triangles).

In keeping with methods employed in previous studies of pentastomid morphology, we plotted raw AD (overall hook length) against BC (depth of hook shank) of posterior hooks to visualize distinct clusters, which are generally indicative of separate species (Fig. 6a). Four potential clusters were initially visible in our raw data (Fig. 6a), though when we included body size as a covariate, two of these clusters combined (leaving three clusters: Fig. 6b). However, when we applied our molecular species groupings to our morphological data there was no correspondence between the two (Figs. 7 and 8), indicating that hook measurements alone are unreliable for distinguishing between species of the genus *Waddycephalus*. We compared our raw measurements of AD and BC of posterior hooks with those taken on 28 *Waddycephalus* spp. measured by Riley and Self (1981b). Corresponding sex and body size data were not available in Riley and Self (1981b) so our analyses did not correct for either of these variables. Measurements of AD of posterior hooks given in Riley and Self (1981b) were significantly larger overall than in the current study (*F*_1,52_ = 11.92, *P* = 0.001; Fig. 8). BC of posterior hooks did not differ between the two studies (*F*_1,52_ = 2.88, *P* = 0.096; Fig. 8). The majority of hook dimensions of the *Waddycephalus* spp. measured in the present study were smaller than in previously described species of *Waddycephalus* (Fig. 8). Overall, our individuals did not cluster well with previous species groupings and instead, were scattered across clusters, with two conspicuous outliers (Fig. 8). There was substantial overlap in hook morphology between different molecular species groupings (Fig. 8, Table 4). One individual from *S. cucullatus* (Fig. 3: 42 SCUC 2473) conformed closely to the hook morphology of *Waddycephalus superbus*, however this individual falls into *Waddycephalus* sp. 5 (Fig. 3), the remainder of which do not cluster morphologically around *W. superbus* (Figs. 7 and 8). Indeed, one individual from *Waddycephalus* sp. 5 has almost identical posterior hook morphology to the single individual that comprised *Waddycephalus* sp. 2 (Figs. 3 and 8), reiterating that hook morphology is not a reliable characteristic for distinguishing species of the genus *Waddycephalus* (Table 4).

#### 3.3.3 Voucher specimens

Voucher specimens are lodged in the Australian National Wildlife Collection, CSIRO Ecosystem Sciences, Canberra. Accession numbers are as follows: *R. orientalis*: P140–144; *Waddycephalus* sp. 2: P153; *Waddycephalus* sp. 3: P155–157; *Waddycephalus* sp. 4: P154, *Waddycephalus* sp. 5: P148–151.

## 4 Discussion

Overall, 59% of the tropical Australian snakes that we dissected were infected with at least one species of pentastomid. The pentastomids were of the genera *Raillietiella* and *Waddycephalus* and infected a range of host taxa, encompassing seven snake species (*S. cucullatus*, *T. mairii*, *D. punctulatus*, *A. praelongus*, *D. vestigiata*, *D. papuensis*, and *L. fuscus*) from three snake families (Colubridae, Elapidae, and Pythonidae). Prevalence of infection was highest in the terrestrial elapid *D. vestigiata* (100%) and in the arboreal colubrid *D. punctulatus* (79%). All of the seven snake species that were infected represent new host records for pentastomids of the genera *Waddycephalus* and/or *Raillietiella*. We found no pentastomids infecting the colubrids *Boiga irregularis* and *Enhydris polylepis*, nor the python *Antaresia childreni* (*n = *3, 1, 4 respectively). Given small sample sizes for these three taxa, an apparent absence of pentastomids may not reflect a lack of infection for the taxa overall.

Two additional genera of pentastomids known to infect Australian snakes were not encountered during our surveys of snakes in the tropics of the Northern Territory: *Parasambonia* and *Armillifer*. The genus *Parasambonia* is unique to Australia and includes two species: *Parasambonia bridgesi* and *Parasambonia minor* (Riley and Self, 1982). *Parasambonia bridgesi* has been recorded in the elapids *Demansia psammophis, Pseudechis porphyriacus*, and *Tropidechis carinatus* in Queensland and New South Wales, and *P. minor* has been recorded in the elapid *Austrelaps superbus* from unknown localities (though this snake occurs only in Victoria and Tasmania; Riley and Self, 1982). Considering that we did not detect any pentastomids of the genus *Parasambonia*, and that the only known hosts occur on the east coast of Australia, it is plausible that the genus *Parasambonia* is not present in the Northern Territory. The other genus that was not represented in our surveys was *Armillifer*, a genus that is represented by two species in Australia: *Armillifer australis* and *Armillifer arborealis* (Riley and Self, 1981a). This genus is responsible for many human cases of pentastomiasis (Fain, 1975; Drabick, 1987). *Armillifer australis* is known only from large Australian pythons (*Liasis olivaceus*, *Morelia amethistina*, *Morelia spilota*, and *Morelia viridis*) from Queensland, and *A. arborealis* has been recorded in *B. irregularis* from Darwin (Riley and Self, 1981a). Due to a lack of available specimens, our survey omitted the only two known hosts for *Armillifer* (*L. olivaceus* and *M. spilota*) that occur at our study site, and included only three individual *B. irregularis*. Therefore, both species of *Armillifer* may well be present but were not represented in our survey.

All snakes sampled were road-killed animals. If pentastomes influence snake respiration and consequently locomotion, infected snakes may be more susceptible to being run over by motor vehicles (Wilson et al., 2002), and thus, our sampling method might have overestimated pentastome prevalence. Even so, the ubiquity of pentastomid infections in snakes of the Australian tropics sampled in this study is perplexing, considering the often-adverse consequences of infection and the recognized zoonotic potential of these parasites (Fain, 1975; Paré, 2008; Yao et al., 2008; Tappe et al., 2011). The large body sizes and high infection intensities (up to 65 mm, up to 77 pentastomids, respectively) attained in the current study reveal that the biomass of pentastomid infections can be substantial, significantly reducing the volume of the lung lumen and occluding respiratory passages, thus reducing host aerobic capacity. *Waddycephalus* spp. in particular were very large relative to the lungs they inhabited, and pentastomid body size did not vary with host species, therefore slender hosts (such as tree snakes) may be particularly affected. Future studies could usefully evaluate the effects of these pentastomes on host performance.

Pentastomids were recovered from the mouth, trachea, lungs, and air sacs of dissected snakes; however for *Waddycephalus* infections, large lesions (indicating sites where pentastomids had attached and fed) were only observed in lung tissue. Because the specimens that we examined were road-killed, it is likely that pentastomids discovered in the mouth and trachea moved there post-mortem. Pentastomids often exit the lungs of sick or dying hosts (*e.g.*, Montgomery et al., 2006; Paré, 2008) and in one instance we observed a large *R. orientalis* crawling out of the mouth of its dead *D. vestigiata* host (see Fig. 1c). Other than the lesions noted above, we did not assess the pathology associated with infections in these snakes. Several case reports describe snake death and disease in association with pentastomiasis. Necroscopy of a deceased Nigerian royal python (*Python regius*) revealed adult pentastomids (*Armillifer* sp.) in the lungs, and suspected larval pentastomids calcified within the liver (Ayinmode et al., 2010). The death of four Dominican boa constrictors (*Boa constrictor nebulosa*) was attributed to infection with *Porocephalus dominicana* (Riley and Walters, 1980). Pneumonia in Gaboon vipers (*Bitis gabonica*) has been linked to infection with *A. armillatus* (Jacobson, 2007). Severe pulmonary tissue damage was reported in a Brazilian colubrid snake (*Philodryas nattereri*) with a heavy pentastomid (*Cephalobaena tetrapoda*) infection (Almeida et al., 2008).

*Raillietiella orientalis* occurred in the elapids *A. praelongus*, *D. papuensis*, *D. vestigiata*, the colubrids *T. mairii* and *D. punctulatus*, and the python *L. fuscus*. It was most prevalent in *D. vestigiata*, with 100% of snakes infected (*n = *19). Infection intensities ranged from 1 to 77 per infected host and severe occlusion of the lung was apparent in those snakes on the higher end of the intensity spectrum. The scarcity of ecological studies on *R. orientalis* makes it difficult to discern whether the levels of infection we encountered in Australian snakes are similar to those in other regions. Norval et al. (1982) reported that 4 of 6 mountain wolf snakes (*Lycodon ruhstrati*) were infected with *R. orientalis* in Taiwan, with intensity ranging from 2 to 59 pentastomids per infected host.

Body size of *R. orientalis* varied with pentastome sex and with host species. Females were longer than males and pentastomids from *T. mairii* were smaller than those from *D. vestigiata*. This may reflect differences in host size: *T. mairii* is smaller than *D. vestigiata* (Greer, 1997) and consequently, lung size should differ accordingly. Host-dependent parasite morphology has been reported in *R. frenata*, with pentastomes reaching larger body sizes in the gecko *H. frenatus* than in the toad *R. marina* (Kelehear et al., 2011). In this instance morphology was not dependent on lung size. Our measurements of key morphological characteristics distinguishing species of the genus *Raillietiella* indicated that morphology of *R. orientalis* differs between Australia and Asia. Specifically, AB of posterior hooks and copulatory spicules were larger in the present study than in Ali et al. (1982), highlighting the need to look beyond morphological measurements alone to identify species. Further, the relationship between pentastome body size and hook size varied depending on how many covariates were included in the analysis. The most basic analysis (pentastome body length *vs* hook size) showed a positive correlation between body size and AB and BC of anterior and posterior hooks (*R*^2^ = 0.69–0.82), reiterating the importance of considering pentastomid body size in morphological comparisons between species of the genus *Raillietiella* (see also Kelehear et al., 2011).

*Raillietiella orientalis* has previously been recorded from the snake families Colubridae, Elapidae, Viperidae, and Pythonidae in Asia (Ali et al., 1982). Recently, we found three specimens in two cane toads (*R. marina*) at our study site in the tropics of the Northern Territory, Australia (Kelehear et al., 2011). Only two of 2779 cane toads were infected with *R. orientalis* in those studies (CK pers. obs.), implying that *R. marina* is an incidental host and may occasionally acquire infections when feeding at infected *D. vestigiata* carcasses (Kelehear et al., 2011). Birds of prey commonly feed on road-killed carcasses; in particular, black kites (*Milvus migrans*) and whistling kites (*Haliastur sphenurus*) are common at our study site, where they feed on carcasses of *Demansia* spp., and presumably other snakes also (CK pers. obs.). Recently, pentastomes were recorded in the air sacs of two terrestrial birds, both of which are scavengers: a black vulture (*Aegypius monachus*) infected with *Hispania vulturis* (a newly erected genus of pentastome) in Spain (Martínez et al., 2004), and an oriental white-backed vulture (*Gyps bengalensis*) infected with *Raillietiella trachea* in Pakistan (Riley et al., 2003). The authors hypothesized that these pentastomids possessed a direct life cycle; nonetheless, the scavenging habits of these hosts, and the fact that the oriental white-backed vulture was infected with a raillietiellid, suggest that these parasites may have been ingested with prey. The potential for scavengers to become infected with pentastomids via this pathway warrants further research.

The life cycle of *R. orientalis* is unknown but has been hypothesized by Ali et al. (1982) to include one or more lizard and/or snake intermediate hosts. Presumably this parasite entered Australia from Asia when an infected intermediate host was accidentally introduced. When considering potential intermediate hosts for trophically transmitted parasites, such as pentastomids, it is imperative to consider the diet of the hosts, especially those with specialized diets. Of the ∼20 known ophidian definitive hosts of *R. orientalis*, few have specialized diets. One that does is the highly aquatic Asiatic water snake (*Xenochrophis piscator*) that primarily eats fish (>75% of diet) and to a lesser extent, frogs (>14% of diet; Brooks et al., 2009). None of 232 Asiatic water snakes examined by Brooks et al. (2009) contained a snake in their stomach contents. Therefore, snakes are an unlikely intermediate host for *R. orientalis*. Snakes are not commonly consumed by any of the definitive host species from which we recovered *R. orientalis*. Fish are not consumed by *Demansia* spp. which exhibited 100% prevalence of infection, hence it is unlikely that they are the primary intermediate host for *R. orientalis*. Frogs are the most plausible intermediate host for this pentastomid. All snake species that were infected with *R. orientalis* consume frogs (Greer, 1997) and *T. mairii* preys almost exclusively on frogs (Shine, 1991a). We detected only one *R. orientalis* in one individual of the arboreal *D. punctulatus* which preys primarily on tree frogs in the Northern Territory (Shine, 1991a). Thus, a ground dwelling frog is the most plausible intermediate host for *R. orientalis* in the Australian tropics. We found one *R. orientalis* in one water python (*L. fuscus*). These pythons feed primarily on native rats, *Rattus colletti*, at the only site where this species has been studied intensively (close to our own study area; Madsen et al., 2006). However, dissections of water pythons from other regions (including nearby) have revealed a broader diet, with significant numbers of avian and reptilian prey (Shine and Slip, 1990). Declines in rat abundance caused by flooding events may induce the pythons to feed upon a wider range of species (Ujvari et al., 2011). Future studies could usefully examine ground dwelling frogs for presence of infective *R. orientalis* larvae.

We detected adult *Waddycephalus* spp. in the elapid *D. vestigiata*, and the colubrids *S. cucullatus*, *T. mairii*, and *D. punctulatus*; the former three species are new host records for pentastomids of the genus *Waddycephalus*. We recovered nymphal *Waddycephalus* sp. from the elapids *A. praelongus* and *D. vestigiata*, both of which are new host records for *Waddycephalus* nymphs. Our molecular results suggest that there are five species of *Waddycephalus* present in the snakes sampled in the Northern Territory. We were unable to obtain complete morphological data for all *Waddycephalus* specimens that we sequenced, firstly because hosts were road-killed and specimens were often damaged, and secondly because the chemical used to clear pentastomids for morphological investigation rendered the samples unsuitable for molecular analyses. Because three of our five species of *Waddycephalus* (*Waddycephalus* sp. 1, 2, and 4) were represented by a single pentastomid, these data are too scant to warrant substantial discussion. Instead, we focus on *Waddycephalus* sp. 3 and sp. 5, for which sample sizes were larger.

Adult *Waddycephalus* spp. were most prevalent in *D. punctulatus* (79%), in which infection intensities were as high as 10 pentastomids per infected host. *Dendrelaphis punctulatus* is the type host for *W. punctulatus* described from the east coast of Queensland, Australia (Riley and Self, 1981b) and to date no other pentastomids from this genus have been recorded from this host. We recovered two species from *D. punctulatus*: *Waddycephalus* sp. 3 and *Waddycephalus* sp. 4. *Waddycephalus* sp. 3 occurred primarily in *D. punctulatus*, and to a lesser extent, in *S. cucullatus*; the sole member of *Waddycephalus* sp. 4 infected *D. punctulatus*. The maximum body size of female *Waddycephalus* sp. 3 (42 mm) exceeded that of *W. punctulatus* (36 mm) and male *Waddycephalus* sp. 3 were all smaller (range: 9.6–11 mm) than those from *W. punctulatus* (range: 13–16 mm; Riley and Self, 1981b). Total annuli counts (63 annuli) from the only *Waddycephalus* sp. 3 female we could assess was within the range of annuli number for *W. punctulatus* (Riley and Self, 1981b). However, given the discrepancies in body size and the marked variability in hook size for *Waddycephalus* sp. 3 we do not consider it to be *W. punctulatus*. We were unable to obtain data on body size or annuli for the sole specimen of *Waddycephalus* sp. 4.

*Stegonotus cucullatus* were infected with *Waddycephalus* sp. 3 and sp. 5 at a prevalence of 60%, representing the first records of pentastomids infecting this host species. *Waddycephalus* sp. 5 occurred primarily in *S. cucullatus*, although one individual was recovered from *D. vestigiata*. *Waddycephalus* sp. 5 is a relatively small species with a maximum size of 21 mm. Hook dimensions for this species were again highly variable. Several *Waddycephalus* sp. 5 conformed closely to the hook morphology of *Waddycephalus scutata*, but others varied substantially. The primary distinguishing feature of *W. scutata* is narrow body width (maximum 3.5 mm) which distinguishes it from other small species of *Waddycephalus* whose body width exceeds 5.0 mm (Riley and Self, 1981b). *Waddycephalu*s sp. 5 were relatively thin individuals with a maximum body width of 4.0 mm so (ignoring hook dimensions) this species may be *W. scutata*. Adult *W. scutata* are known from the tiger snake (*Notechis scutatus*) on islands off the coast of South Australia (Riley and Self, 1981b) and from the yellow-faced whip snake (*D. psammophis*) at a Park in the Northern Territory (Poore and Spratt, 2012). The latter authors also reported nymphs from the northern quoll (*Dasyurus hallucatus*).

The life cycles of species in the genus *Waddycephalu*s are yet to be elucidated; frogs, lizards or mammals are likely intermediate hosts (Riley and Self, 1981b). The terrestrial and arboreal snakes infected with *Waddycephalus* spp. in the current study all prey primarily on frogs and lizards (Shine, 1980, 1991a; Greer, 1997; Fearn and Trembath, 2009, 2010; Trembath et al., 2009; Kelehear, 2012a,b), implying an ectothermic intermediate host for *Waddycephalus*. Our dissections revealed three *Waddycephalus* nymphs, two in the lungs of *D. vestigiata*, and one attached to the exterior surface of the lungs in *A. praelongus*. One *Waddycephalus* nymph has also been recovered from one *H. frenatus* near our study area (Barton, 2007). Nymphs of *Waddycephalus* spp. have been recovered from diverse taxa in other Australian localities (Online Supplementary Table 1), including dasyurid marsupials, a sooty owl (*Tyto tenebricosa*), a small-eyed snake (*Cryptophis nigrescens*), a Bynoe’s gecko (*Heteronotia binoei*), and a three-toed earless skink (*Hemiergis decresiensis*). *Waddycephalus* nymphs were also recovered from the remote froglet (*Crinia remota*) in Papua New Guinea (Riley and Spratt, 1987). The nymphs were in diverse localities within the hosts and were often encapsulated, implying that either they were infective and waiting to be consumed by a definitive host to complete maturation, or that they had entered accidental hosts and would progress no further in their life cycle. Future research is needed to elucidate the life cycles of *Waddycephalus* pentastomids.

Clearly, substantial taxonomic work remains to be done on pentastomids in Australia – particularly with respect to the genus *Waddycephalus*. Future work should employ a combination of molecular and morphological techniques and aim to unearth morphological characteristics that may be useful for species identification.

## Figures and Tables

**Table 1:** Native Australian snakes collected in the tropics of the Northern Territory and inspected for pentastomids (*Waddycephalus* spp. and *Raillietiella orientalis*) between November 2008^∗^ and July 2011.

Host	*n*	*Raillietiella orientalis*			*Waddycephalus* spp.			Host foraging mode	Host diet†
		Prevalence (%)	Mean intensity ± 1 S.E.	Max. intensity	Prevalence (%)	Mean intensity ± 1 S.E.	Max. intensity		
*Boiga irregularis*	3	0.00	–	–	0.00	–	–	Aboreal & terrestrial	Birds (36%), reptiles (35%), mammals (23%), frogs (6%)
*Dendrelaphis punctulatus*	14	7.14	1	1	78.57	3.9 ± 1.1	10	Aboreal	Frogs (78%), reptile eggs (19%), mammals (3%)
*Stegonotus cucullatus*	10	0.00	–	–	60.00	4.7 ± 0.7	7	Aboreal & terrestrial	Reptiles (72%), reptile eggs (17%), mammals (11%)
*Tropidonophis mairii*	12	41.67	2.8 ± 1.4	8	8.33	1	1	Terrestrial & aquatic	Frogs (97%), fish (1%), reptile eggs (1%), mammals (1%)
*Enhydris polylepis*	1	0.00	–	–	0.00	–	–	Aquatic	Fish (70%), frogs (30%)
*Acanthophis praelongus*⁎	10	40.00	3.8 ± 1.7	8	10.00§	1§	1§	Terrestrial	Frogs (39%), reptiles (31%), mammals (30%)
*Demansia papuensis*	1	100.00	8	8	0.00	–	–	Terrestrial	Reptiles (73%), frogs (27%)
*Demansia vestigiata*	19	100.00	12.8 ± 5.0	77	21.05	1.3 ± 0.3	2	Terrestrial	Reptiles (71%), frogs (29%)
*Antaresia childreni*	4	0.00	–	–	0.00	–	–	Aboreal & terrestrial	Mammals (37%), frogs (33%), reptiles (26%), birds (5%)
*Liasis fuscus*	7	14.29	1	1	0.00	–	–	Terrestrial	Mammals (60%), birds (23%), reptiles (17%)

⁎ One *A*. *praelongus* was collected in April 2007.

§ *Waddycephalus* sp. detected in *A. praelongus* was a nymph.

† Dietary data from Shine (1991b), Webb et al. (2005), Fearn and Trembath (2009).

**Table 2:** Between-species *COX1* genetic distances for pentastome species. WSP1–5 = *Waddycephalus* species 1–5; RFRE = *Raillietiella frenata*; RORI = *Raillietiella orientalis*. All pentastomids were from Australian snakes except *R. frenata* which were from the cane toad, *Rhinella marina*. Distances were calculated using MEGA v5.10 with the Kimura 2 Parameter model.

	WSP1	WSP2	WSP3	WSP4	WSP5	RFRE
WSP2	0.053					
WSP3	0.104	0.068				
WSP4	0.159	0.121	0.096			
WSP5	0.104	0.077	0.086	0.129		
RFRE	0.471	0.439	0.411	0.442	0.436	
RORI	0.433	0.400	0.418	0.472	0.424	0.212

**Table 3:** Measurements of *Raillietiella orientalis* recovered from snakes in the Northern Territory of Australia. Minimum–maximum (mean ± 1 S.E.).

*n*	Sex	Body length (mm)	Body width (mm)	AB anterior hook (μm)	BC anterior hook (μm)	AB posterior hook (μm)	BC posterior hook (μm)	Spicule length (μm)	Spicule width (μm)
15	F	9.2–65.2 (42.0 ± 4.3)	0.2–2.4 (1.3 ± 0.2)	208–350 (319 ± 12)	228–424 (376 ± 16)	224–450 (386 ± 18)	270–520 (458 ± 20)	—	—
16	M	4.0–16.5 (9.4 ± 1.2)	0.5–1.7 (1.0 ± 0.1)	73–200 (150 ± 12)	104–250 (187 ± 13)	83–239 (199 ± 12)	141–292 (240 ± 13)	900–1600 (1237 ± 76)	371–1500 (627 ± 86)

**Table 4:** Measurements of *Waddycephalus* spp. recovered from snakes in the Northern Territory of Australia. Minimum–maximum (mean ± 1 S.E.).

Molecular species	*n*	Sex	Body length (mm)	Body width (mm)	# annuli	# post-vaginal annuli	AD anterior hook (μm)	BC anterior hook (μm)	AD posterior hook (μm)	BC posterior hook (μm)
*Waddycephalus* sp. 2	1	M	7.2	1.2	Unk.	—	477	318	530	329
*Waddycephalus* sp. 3	8	F	15.1–42.0 (29.0 ± 6.8)	2.1–5.5 (3.6 ± 0.9)	63⁎	8, 9§	440–900 (664 ± 58)	270–530 (410 ± 37)	440–954 (684 ± 62)	290–557 (417 ± 40)
*Waddycephalus* sp. 3	4	M	9.6–11.0 (10.3 ± 0.3)	0.3–2.2 (0.9 ± 0.4)	55, 60§	—	350–477 (426 ± 39)	265–450 (338 ± 57)	340–530 (449 ± 57)	318–480 (399 ± 47)
*Waddycephalus* sp. 4	1	F	Unk.	Unk.	Unk.	Unk.	640	370	690	400
*Waddycephalus* sp. 5	6	F	18.1–21.2 (19.7 ± 1.6)	2.1–4.0 (3.0 ± 1.0)	Unk.	Unk.	398–848 (698 ± 150)	318–530 (442 ± 64)	530–981 (804 ± 139)	451–557 (513 ± 32)
*Waddycephalus* sp. 5	3	M	7.2–12.5 (9.8 ± 1.5)	1.2–2.4 (1.9 ± 0.4)	Unk.	—	424–477 (459 ± 18)	292–334 (315 ± 12)	451–530 (502 ± 26)	334–371 (359 ± 12)
*Waddycephalus* sp. 5	1	Nymph	6.2	0.8	Unk.	Unk.	Unk.	Unk.	Unk.	Unk.
*Waddycephalus* sp. unk.	3	F	25.2–38.2 (31.7 ± 6.5)	4.8–5.0 (4.9 ± 0.1)	65	7, 7§	900–1007 (953 ± 54)	530–530 (530 ± 0)	954–1060 (1007 ± 53)	557–583 (570 ± 13)
*Waddycephalus* sp. unk.	3	M	5.3–14.5 (10.4 ± 2.7)	0.9–1.67 (1.3 ± 0.2)	Unk.	—	318–382 (339 ± 21)	212–292 (242 ± 25)	371–398 (385 ± 14)	239–239 (239 ± 0)
*Waddycephalus* sp. unk.	1	Nymph	5.2	0.8	Unk.	—	239	318	239	471

⁎ *n* = 1.

§ *n *= 2.
