# Supplementary material for: Pentastomids of wild snakes in the Australian tropics
Source: Int J Parasitol Parasites Wildl. 2013 Dec 31;3(1):20–31. doi: 10.1016/j.ijppaw.2013.12.003 (PMC4047961; doi:10.1016/j.ijppaw.2013.12.003)
Supplement: Supplementary data 1 [file mmc1.docx]

**PENTASTOMIDS OF WILD SNAKES IN THE AUSTRALIAN TROPICS**

**Crystal Kelehear, David M. Spratt, Denis O’Meally, and Richard Shine.**

**Online Supplementary Table 1**. Recorded hosts and localities for *Waddycephalus* sp. nymphs taken from the literature and museum records.

| **Host family** | **Host species** | **Location within host** | **Collection locality^a^** | **Reference** |
| --- | --- | --- | --- | --- |
| Dasyuridae | Yellow-footed Antechinus (*Antechinus flavipes*) | Mesenteric and subcutaneous connective tissue | Coutts Crossing, Grafton, NSW | Australian National Wildlife  Collection (W/L HC #P82) |
| Dasyuridae | Brown antechinus (*Antechinus stuartii*) | Anal gland | Glenreagh, via Grafton, NSW | Australian National Wildlife  Collection (W/L HC #P81) |
| Dasyuridae | Dibbler (*Parantechinus apicalis*) | Unknown | Unknown | ([Riley and Spratt, 1987](#_ENREF_2)) |
| Dasyuridae | Common Dunnart (*Sminthopsis murina*) | Mesentery (encapsulated) | Grafton, NSW | Australian National Wildlife  Collection (W/L HC #P77) |
| Dasyuridae | Little Red Kaluta (*Dasykaluta rosamondae*) | Abdomen (encapsulated) | Woodstock and Abydos Stations, via Marble Bar, WA | ([Riley and Spratt, 1987](#_ENREF_2)) |
| Dasyuridae | Northern Quoll (*Dasyurus hallucatus* = *Satanellus hallucatus*)  Northern Quoll (*Dasyurus hallucatus*) | Mesentery  Liver | Nourlangie, NT  Coomalie Creek, NT | ([Riley et al., 1985](#_ENREF_3))  Australian National Wildlife  Collection (W/L HC #P89) |
| Tytonidae | Sooty Owl (*Tyto tenebricosa*) | Mesentery | Mebbin State Forest, NSW | Australian National Wildlife  Collection (W/L HC #P83) |
| Elapidae | Small-eyed Snake (*Cryptophis nigrescens*) | Intestinal connective tissue (encapsulated) | Mogo State Forest, NSW | ([Riley and Spratt, 1987](#_ENREF_2)) |
| Elapidae | Lesser Black Whip Snake (*Demansia vestigiata*) | Lungs | Middle Pt, NT | This study |
| Elapidae | Northern Death Adder (*Acanthophis praelongus*) | Attached to exterior surface of lungs | Middle Pt, NT | This study |
| Gekkonidae | Binoe’s Gecko (*Heteronotia binoei*) | Encapsulated | Girraween National Park, Qld | ([Riley and Spratt, 1987](#_ENREF_2)) |
| Gekkonidae | Asian House Gecko (*Hemidactylus frenatus*) | Body wall | Black Point & Berry Springs, NT | ([Barton, 2007](#_ENREF_1)) |
| Scincidae | Three-toed Earless Skink (*Hemiergis decresiensis*) | Lungs (encapsulated) | SA | ([Riley and Spratt, 1987](#_ENREF_2)) |
| Myobatrachidae | Remote Froglet (*Crinia remota* = *Ranidella remota*) | Below post-orbital skin | Papua New Guinea | ([Riley and Spratt, 1987](#_ENREF_2)) |

^a^ All locations are within Australia unless otherwise indicated.

**ONLINE SUPPLEMENTARY TABLE 1 REFERENCES**

Barton, D.P., 2007. Pentastomid parasites of the introduced Asian house gecko, *Hemidactylus frenatus* (Gekkonidae), in Australia. Comp. Parasitol. 74, 254-259.

Riley, J., Spratt, D.M., 1987. Further observations on pentastomids (Arthropoda) parasitic in Australian reptiles and mammals. Rec. South Aust. Mus. 21, 139-147.

Riley, J., Spratt, D.M., Presidente, P.J.A., 1985. Pentastomids (Arthropoda) parasitic in Australian reptiles and mammals. Aust. J. Zool. 33, 39-53.
